# Can new healthy luxury food products accelerate short food supply chain formation via social media marketing in high-income countries?

**DOI:** 10.1186/s40100-022-00238-3

**Published:** 2022-12-09

**Authors:** Christoph F. Wiedenroth, Verena Otter

**Affiliations:** 1 Department of Agricultural Economics and Rural Development, University of Goettingen, Platz der Goettinger Sieben 5, 37073 Goettingen, Germany; 2 Business Management & Organisation Group, Wageningen University, Hollandseweg 1, 6706 KN Wageningen, The Netherlands

**Keywords:** Short food supply chains, Social media marketing, New healthy luxury food products, Food quality perception, Superfoods, Measurement of media trust, Agricultural digitalization

## Abstract

Social media marketing is a promising tool for successful product placement of new healthy luxury food products, a subcategory of superfoods. Despite its growing popularity, no studies have investigated how social media marketing affects consumers’ quality perception process for such superfoods and whether this provides opportunities for farmers to gain a competitive advantage in direct marketing channels. Therefore, we integrate media richness theory into the food quality guidance model, compile a data set of 697 German fruit consumers from May to June 2020, and analyze this sample via partial least square analysis. Results show that social media marketing is a viable tool for new healthy luxury food products if media content is highly experience providing. Furthermore, it offers opportunities for the formation of shorter food supply chains as farmers could, through the provision of engaging social media marketing content, sell new healthy luxury food products directly to the final consumer. This research provides implications to farmers, retailers and policy makers to exploit the social media marketing potential of new healthy luxury food products.

## Introduction

Many traditional food products rich in health-benefiting substances, such as fiber and antioxidants, are nowadays marketed as superfoods (Merriam-Webster 2021). Throughout high- and middle-income countries, the demand for superfoods has grown sharply in recent years with a current market size of USD 164 billion and a projected annual growth rate of 6.77% until the year 2027 (The Business Research Company 2022). This trend arises from superfoods fitting consumers’ improved levels of health awareness as well as their increasing desire to display social class distinction through health-related activities (Graeff-Hönninger and Khajehei 2019; Loyer 2016). Such activities can include regular exercising and nutrient-rich eating habits (Pampel et al. 2010). Particularly the latter is becoming more and more important to consumers and represents a key marketing characteristic of superfoods in the future (Oude Groeniger et al. 2017). Superfoods are regarded as highly beneficial to health by many consumers and, due to their high product price, as a luxury product that is expensive but still affordable at the same time (Butterworth et al. 2020). Mundel et al. (2017) described such food commodities as ‘affordable luxuries’ for which consumers hold similar quality expectations to traditional luxury products. This health-related luxury dimension of superfoods is likely to become even more important in the future as superfoods gradually substitute former luxury products, such as digital hardware and clothing, which have become more accessible to a wider range of consumer segments (Oude Groeniger et al. 2017). As a result of these different luxury dimensions, some superfoods, such as blueberries, are considered to represent the subcategory of so called new healthy luxury food products (NHLFPs)^1^(Wiedenroth and Otter 2021b).

Particularly for luxury products, well-tailored marketing strategies are crucial as they assist consumers in communicating and comparing unique product experiences associated with their brand, price, and quality criteria (Atwal and Williams 2017). The sharp increase in social media marketing (SMM) expenditures on non-food-related luxury brands, such as car manufacturers, has already disrupted conventional marketing strategies and is continuing to emphasize social media platforms as an important tool for future product placement (Elliott 2019; Godey et al. 2016). SMM’s importance is likely to grow even further as social media platforms are becoming increasingly valuable for developing opinions and influencing behaviors, especially among young people (Liu et al. 2021). Therefore, to marketers of luxury products, social media platforms provide multiple advantages compared with previous marketing strategies. On the one hand, they assist consumers in individualizing and in comparing their actions with friends more easily. This is highly beneficial to luxury product marketing as consumers’ ability to engage and to compare their shopping activities is a central consumption motive of luxury products. On the other hand, social media platforms offer a unique selling point to retailers because they enable targeted group advertising (Arrigo 2018; Kim and Ko 2010; Liu et al. 2021). Similar or even stronger benefits are to be expected for NHLFPs because promising consumer segments are receptive to targeted marketing strategies and regularly share eating habits with friends (Wiedenroth and Otter 2021b; Gunarathne et al. 2017; Hemmerling et al. 2016). Some NHLFP producer associations, such as blueberry growers (FruitGrowerNews 2018), have begun to recognize such developments and started to engage in SMM increasingly. Yet, most of their marketing efforts seem to neglect important NHLFP luxury dimensions that are critical drivers to NHLFP consumption. Developing a better understanding of how SMM can promote different NHFLP luxury dimensions could help steering farmers’ shared NHLFP marketing efforts more efficiently (Wiedenroth and Otter 2021b).

Current sustainability and healthy eating trends, which are particularly distinct among NHLFP consumer groups (Wiedenroth and Otter 2021b), are often compared through food-related activities via social media platforms (Hemmerling et al. 2016). While doing so, consumers tend to value closer connections to the primary producers as the environmental and social impacts of the products that they consume become more visible and thus can be compared more easily (Elghannam et al. 2018, 2020). Such consumer preferences provide opportunities for farmers to gain a competitive advantage via SMM through establishing direct marketing channels. In other subsectors of the agricultural industry, consumers’ desire to engage directly with food producers has already resulted in an increasing formation of short food supply chains (SFSCs) (Giampietri et al. 2018).^2^ Social media platforms are proving to be particularly valuable in promoting this trend (Elghannam et al. 2018, 2020) as they offer several benefits to producers and consumers alike. Producers benefit from low-cost marketing opportunities and easy distribution of information, while consumers can interact with producers more effortlessly and gain simplified access to product-related information (Elghannam et al. 2018, 2020). In addition, both sides benefit from the fact that, through SFSCs, the strong oligopoly formation among companies in the food retail sector, which characterizes the supply chain of agricultural goods in many high-income countries (Dobson et al. 2003), can be circumvented (Elghannam et al. 2020; Shepherd et al. 2020). In the case of NHLFPs, these benefits are likely to be even greater because NHLFPs are already susceptible to SMM and SFSCs support socially comparable actions as luxury dimensions, such as environmental and social sustainability, become more visible (Carbone 2017). To draw conclusions on the role of NHLFP SMM for the development of SFSCs, an in-depth analysis of the consumers NHLFP quality perception process in the context of the digital age is inevitable (Cicatiello 2020; Cicia et al. 2021). The results of such research grant detailed information to farmers and cooperatives on how to design SMM strategies for their NHLFP and to agricultural consultants and policy makers on how to assist farmers in promoting SFSC formation through SMM. In particular, among policy makers supporting SFSC formation has gained attention recently, as such chains show higher resilience towards external shocks such as the COVID-19 pandemic (Thilmany et al. 2021).

Despite the relevance of SMM to the marketing of NHLFPs and the opportunities that SMM can offer to primary producers for supplying alternative marketing channels and thus revenue streams, research has not yet adequately addressed this topic. Until now, scholars have only confirmed the impact of SMM on unhealthy food consumption and its potential for raising the awareness of sustainable product attributes among luxury food consumers in high-income countries (Sogari et al. 2017). Although there has been increased recognition of the growing importance of social media platforms for food marketing (Gunarathne et al. 2017; Hemmerling et al. 2016; Wiedenroth and Otter 2021b), no study has investigated whether SMM is an appropriate tool for marketing NHLFPs, to our knowledge. In addition, few studies have discussed the potential of SMM for building SFSCs (Elghannam et al. 2018, 2020; Shepherd et al. 2020), while no study has considered it with respect to NHLFPs.

Against this background, we raise the following research questions. First, what effect do social media platforms have on consumers’ quality perception of NHLFPs? Second, how can these effects accelerate short food supply chain formation in Germany?

This study consists of the following consecutive sections. Sections 2 and 3 offer a detailed presentation of the theoretical background and conceptual framework. This framework integrates media richness theory into the food quality guidance model for the first time (Steenkamp 1989; van Trijp and Steenkamp 2005) to conceptually consider new food marketing communication trends. These chapters are followed by a description of the data collection process and applied methodology in Sect. 4 Furthermore, Sect. 4 motivates the applied case under research. Finally, the results, a discussion, and the conclusion are presented in Sects 5, 6, and 7, respectively.

## Theoretical background

### Food quality guidance model

High-quality expectations drive the demand for superfoods and are even more distinct among NHLFP consumers (Butterworth et al. 2020; Wiedenroth and Otter 2021b). The quality guidance model introduced by Steenkamp (1989) and van Trijp and Steenkamp (2005) takes this into consideration by providing a framework that reflects the development process of consumers’ food quality perception. The authors outlined four different channels (quality cues) through which quality perceptions can be formed. These different quality cues are subdivided into *intrinsic product attributes*, such as color and texture, which are part of the product itself, and *extrinsic product attributes*, such as price and country of origin, which belong to the product but are ‘physically not part of it’ (van Trijp and Steenkamp 2005, 105). *Experience* and *credence product attributes* represent the third and fourth quality cues. The former describes product characteristics such as taste and texture, which are experienced upon consumption, while the latter includes product features that are unobservable by the final consumer even after consumption (e.g., products’ health benefits and the environmental production impact) (Lee and Hwang 2016; Nelson 1970; van Trijp and Steenkamp 2005, 104). The different quality cues are of varying importance for influencing consumers’ quality perception (Steenkamp 1989). Their ultimate effect is closely linked with consumers’ product observation as well as the external communication of different product features. Consumers’ product observation primarily influences the perception of intrinsic product features and experience attributes, while communication efforts, such as product marketing, mostly influence the perception of credence attributes and extrinsic quality cues (Luning, Marcelis, and Jongen 2002; van Trijp and Steenkamp 2005). Nevertheless, since the introduction of this framework, communication has become much more complex as the development of SMM has introduced new marketing possibilities. Until now, this diversification has not been taken adequately into consideration, causing a considerable shortcoming of this theory as different communication cues are likely to influence perceptions of food attributes differently.

### Media richness theory

**Fig. 1:**
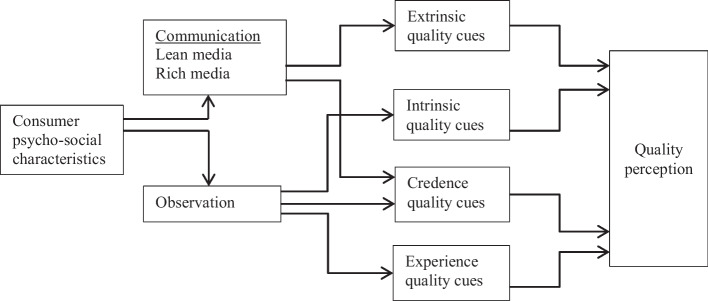
Research framework integrating the food quality guidance model and media richness theory. *Source*: Own elaboration based on Luning, Marcelis, and Jongen (2002)

We integrate the media richness theory into the food quality guidance model to account for the growing importance of choosing the right media channels when communicating food product attributes to a targeted audience (see Fig. 1). At its core, this theory postulates that efficient communication takes place if the characteristics of individual media channels, here media richness, fits the attributes of a particular assignment that needs to be achieved, here the marketing of different quality cues of NHLFPs (Brunelle 2009; Daft and Lengel 1986). The richness of media channels is generally differentiated with respect to their ability to transfer information based on the following four individual characteristics (Daft and Lengel 1986; Ledford 2012): Can receivers respond to the obtained information? To what degree is it possible in communication to target specific focus groups? Can different information channels, such as video and print, be utilized at the same time? What degree of language alteration (use of different words) is possible within a given message? From these, a degree of media richness results that ranges from face-to-face marketing as the richest media channel, incorporating all four characteristics, to text messages and mass mailings as the marketing sources (Brunelle 2009; Ledford 2012). In detail, lean media sources include mostly traditional media sources, such as print and television ads, radio stations, brochures, and advertisements as well as mass mailings, while rich media sources center on social media platforms, such as networking sites, video platforms, and blogs, but include paid television ads as well (Holt et al. 2015; Ledford 2012; Lipowski and Bondos 2018).^3^ Ultimately, based on the information that they seek, consumers will choose different media channels which provide different abundances of information, when searching for particular product information pre- or post-purchase (Lipowski and Bondos 2018). Thus, from a strategic marketing point of view, media channels must be chosen based on the product attributes to be advertised as well as on the target group to be addressed (Holt et al. 2015). In the case of NHLFPs, media richness theory is highly suitable as consumer segments are likely to utilize a large set of different media channels to obtain and share food-related information (Wiedenroth and Otter 2021b).

## Conceptual framework

**Fig. 2:**
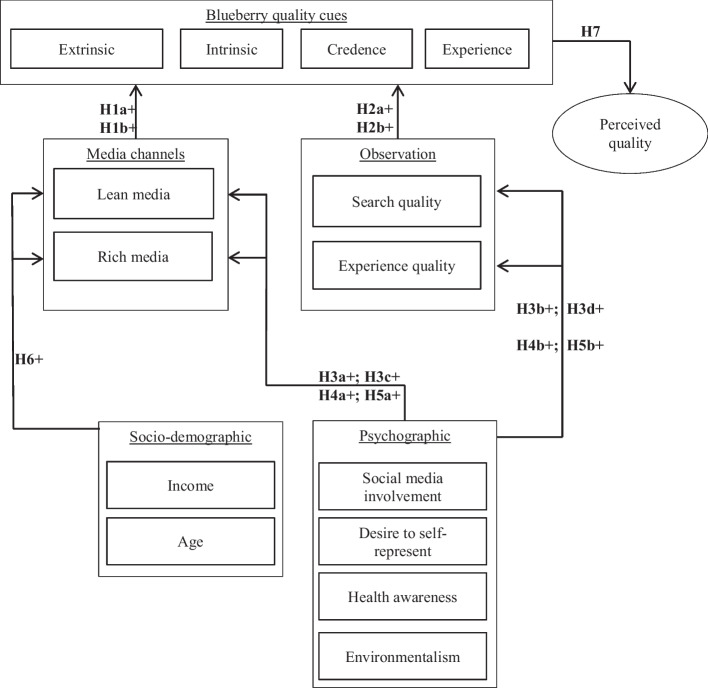
Research model with hypotheses. *Source*: Authors’ own graphic adapted from Luning, Marcelis, and Jongen (2002) and Otter, Prechtel, and Theuvsen (2018)

The conceptual framework that is developed in the following is presented in Fig. 2.

### Media

Research has stressed the differences in the impacts of lean and rich media channels on (food) product quality perceptions (Holt et al. 2015; Ledford 2012). The strong influence of lean media channels on consumers’ food choices has been well established within the literature. For example, radio advertisements and advertisements in children’s magazines can positively influence unhealthy food choices (Smith et al. 2019). A similar relationship is expected for NHLFP consumption as lean media channels often serve as an important food-related information source for corresponding consumer segments (Wiedenroth and Otter 2021b). Therefore we assume, in line with the propositions of Degeratu, Rangaswamy, and Wu (2000) and Lipowski and Bondos (2018), that lean media channels will positively influence the perception of extrinsic and credence quality cues.

Concerning rich media channels, marketing channels other than social media platforms have also been linked to a change in consumers’ food quality perception. For example, television ads influence the brand attachment of food products and lead to a change in consumers’ credence and intrinsic quality attribute awareness (Boyland et al. 2016; Cairns 2019; Kelly et al. 2019). Likewise, and in line with our research objective, similar observations have been made for social media platforms (Dunlop, Freeman, and Jones 2016). Across social media platforms, marketing strategies such as influencer marketing can strongly affect unhealthy (Boyland et al. 2016; Coates et al. 2020) and often weakly influence healthy (Williams et al. 2014) eating habits among adults as well as children. Familiarity with social media platforms is also likely to influence consumers’ receptiveness to intrinsic food quality cues as different social media trends have affected the importance that consumers place on food colors (Southey 2019). Furthermore, it can raise consumers’ awareness of credence food quality attributes, such as environmentally sustainable production practices (Sogari et al. 2017). Therefore, SMM strategies can clearly influence consumers’ attitude toward intrinsic quality cues as well as the credence quality attributes of food products. In the case of traditional luxury products, SMM has been found to lead to higher brand awareness and improved brand image, thereby persuading and engaging consumers more strongly, which highlights an impact on extrinsic quality cues (Chu, Kamal, and Kim 2013; Kim and Ko 2010).

#### H1a

Lean media channels will positively influence perceptions of extrinsic quality cues and credence quality attributes.

#### H1b

Rich media channels will positively influence perceptions of extrinsic and intrinsic quality cues as well as credence quality attributes.

### Observation

Concerning consumers’ product observation, Nelson (1970) subdivided this process into consumers’ search and experience qualities. Search qualities are needed for evaluating product attributes such as color and price, which can be determined before purchase, while experience qualities result from previous consumption. Hence, experience qualities develop only after purchasing a given food product (Ford, Smith, and Swasy 1988). Both qualities are relevant to our research case (Butterworth et al. 2020), as some of the described luxury dimensions of NHLFPs are purely accessible through consumers’ search qualities (e.g., product price) while non-observable luxury attributes (e.g., health benefits) can only be examined through experience qualities. In particular, the latter is likely to influence perceptions of NHLFP quality cues meaningfully. As NHFLP health-related luxury dimensions are not intuitively visible, easy utilization of these luxury dimensions for socially comparable activities is not possible. To address this problem, consumers build on the credence-related extrinsic, intrinsic, and experience quality attributes of NHLFPs (Southey 2019). For example, they purchase brightly colored superfoods as other consumers associate these colors with an above average health benefit (Southey 2019). However, utilizing the right quality cues, such as accepting a given bitterness in taste or purchasing brightly colored food, both synonyms for particularly health products, will certainly be experience related. At the same time, search qualities are important for identifying traditional food product luxury dimensions, such as the product price.

The literature has determined that the existing levels of consumers’ search and experience qualities influence their product quality perception (Migliore et al. 2015). Concerning consumers’ search qualities, the link with respective product quality cues is not as well established. However, higher search qualities have been found to influence consumers’ evaluation of intrinsic quality cues and experience-related product attributes (Sogn‐Grundvåg and Østli 2009). Consumers’ experience of quality, on the other hand, can influence all four described quality cues of food products (Migliore et al. 2015; Schmitt 1999). In particular, past experience is strongly linked to the degree of importance that consumers attribute to intrinsic and extrinsic product quality cues (Frez Muñoz, Steenbekkers, and Fogliano 2016; Schmitt 1999). Furthermore, when investigating buyers’ behavior in the case of fruit products more closely, Migliore et al. (2015) found that their product experience influences their perception of credence and quality attributes.

#### H2a

Search and experience qualities will positively influence perceptions of extrinsic and intrinsic quality cues.

#### H2b

Search and experience qualities will positively influence perceptions of credence and experience quality attributes.

### Psychographic characteristics

Previous research (Wiedenroth and Otter 2021b) has identified consumers’ involvement with social media platforms as well as their health and environmental awareness as strongly distinguishing NHLFP consumer groups. High involvement with social media platforms will, by definition, lead to intensified usage of social media platforms. At the same time, higher social media involvement is unlikely to substitute the utilization of lean media channels substantially (Kilian, Hennigs, and Langner 2012). Rather, consumer segments with high social media involvement have been found to be surprisingly heterogeneous, and high levels of lean media usage can simultaneously be present (Kilian, Hennigs, and Langner 2012). Involvement with social media platforms is also likely to influence consumers’ food product observation. For example, consumers have been found to inform themselves extensively about food attributes through social media platforms (Hemmerling et al. 2016), which makes it likely that these platforms will influence NHLFP observational criteria, too (Wiedenroth and Otter 2021b). In addition, consumers’ desire to self-represent and to compare their activities with those of others is an essential driver of social media platform utilization (Alhabash and Ma 2017; DeVito, Birnholtz, and Hancock 2017). Thus, consumers who show a high tendency to engage in socially comparable activities are also likely to use social media platforms more intensively.

#### H3a

Higher involvement with social media will positively influence the degree of rich media channel utilization.

#### H3b

Higher involvement with social media will positively influence search and experience qualities.

#### H3c

Consumers’ desire to self-represent will positively influence their degree of rich media channel utilization.

#### H3d

Consumers’ desire to self-represent will positively influence search and experience qualities.

The positive relationship between health awareness and healthy food choices, especially for fruits, has been well established (Giampietri et al. 2021). In addition, health awareness is likely to influence the degree to which consumers gather health-related information positively, like the health benefits of particular food products. Consumers’ accumulation of more information will affect their level of experience with a given food product as well as their product examination (search qualities). During the process of acquiring information, consumers access rich and lean media channels to different degrees, younger consumers accessing social media platforms more often (Kempen et al. 2012). This fits an emerging body of literature that has highlighted the importance of social media channels to consumers for acquiring and sharing health-related information (Zhao and Zhang 2017).

#### H4a

Health awareness will positively influence the degree of lean and rich media channel utilization.

#### H4b

Health awareness will positively influence consumers’ search and experience qualities.

Environmental awareness usually describes consumers’ attitudes toward environmentally sensitive consumption and living practices (do Paço et al. 2013; Roberts 1996). Media cues provide consumers with the opportunity to inform themselves about environmental issues, and lean and rich media cues differ in their coverage of environmental topics (Stoddart and MacDonald 2011). In particular, social media platforms are becoming a central tool for addressing consumers’ environmental awareness (Sumit, Swapnil, and Archana 2018, p. 117ff.). This is because social media platforms provide a greater depth of information and an environment in which likeminded social networks can develop and topics centered on environmental sustainability issues are widely shared (Williams et al. 2014). Although the influence of environmental awareness on social media usage has not received much attention until now, we expect a positive relationship to exist. People with higher awareness levels are often more willing to share their experience and expertise with others, which drives them to use social media platforms, as social networks can be tied there more easily (Johnson et al. 2014; Loebnitz et al. 2015). In addition, environmental awareness has been found to influence consumers in their product evaluation as more attention is paid to extrinsic and experience quality attributes (Loebnitz et al. 2015). This shift is likely to be observable through the changing search and experience qualities of these consumers and thus should be considered in this framework.

#### H5a

Environmental awareness will positively influence the degree of lean and rich media channel utilization.

#### H5b

Environmental awareness will positively influence search and experience qualities.

### Socio-demographic characteristics

Among consumers’ socio-demographic characteristics, their level of income and their age strongly determine their NHLFP consumer segment affiliation (Wiedenroth and Otter 2021b). Household income has also been identified as an important predictor of lean and rich media usage (Speck and Elliott 1997), in which higher levels of income can lead to greater use of rich media sources, particularly social media platforms (Perrin 2015). At the same time, households’ level of income might positively influence their usage of newspapers, which indicates a positive influence on various lean media channels (Anderson 2018). With regard to consumers’ age, one can observe that younger age groups are less likely to read daily print newspapers than older age groups (television consumption remains almost equally high) and that they access the internet as well as social media platforms significantly more often (Perrin 2015). More importantly, different age groups differ in their perceived media richness of the same media channels, which further motivates the inclusion of age in the research model of this study (Lipowski and Bondos 2018).

#### H6a

Higher income will positively influence the degree of lean and rich media utilization.

#### H6b

Higher age has a negative influence on the degree of rich media utilization.

### Product quality attributes

In line with the framework introduced by Luning, Marcelis, and Jongen (2002), food quality needs to be understood as a multidimensional concept that is influenced by extrinsic and intrinsic quality cues as well as credence and experience quality attributes (see Fig. 2) (Alonso, Paquin, and Mangin 2002).

Extrinsic quality cues, such as the product brand or quality seals, like a product’s country of origin, can positively influence the quality perception of consumers (Otter, Prechtel, and Theuvsen; Silva et al. 2017). Brands and quality seals function as quality indicators and assist consumers in reducing the uncertainty prior to their purchasing decision. Once the product brand has been examined, consumers tend to move on to other extrinsic food product characteristics, such as product price and packaging, for further quality evaluation (Vranešević and Stančec 2003). Both, the product price (Kirchler et al. 2010) and the packaging (Bou-Mitri et al. 2020), can influence the perceived product quality positively.

Intrinsic quality cues impose a substantial influence on consumers’ perceived product quality and their final purchasing decision (Alonso, Paquin, and Mangin 2002). Among fruits, for instance, the product color is positively correlated with an increased consumption level of fruits such as cactus pears (Migliore et al. 2015). However, when it comes to food in general, the influence of intrinsic product characteristics, such as product appearance, on perceived product quality has largely been neglected by research (Symmank 2019); according to our research, this also seems to apply to fruits. Nonetheless, we assume a positive relationship between intrinsic quality cues and perceived food product quality to be present as it fits well with an intuitive understanding of how consumers build food product quality perception.

Credence quality attributes are of particular relevance in the context of NHLFPs, as they describe the luxury dimension that differentiates this product category most strongly from traditional luxury food products. Here, environmental friendliness and health benefits have been identified as important credence attributes for influencing NHLFP quality perceptions (Wiedenroth and Otter 2021b). Both, health benefits (Grunert 2005) and environmental friendliness (Sörqvist et al. 2013), have been found to influence the overall quality perception of other food products positively.

Experience quality attributes of food products, such as their freshness and taste, are important quality determinants of the final consumer (Luning, Marcelis, and Jongen 2002). In particular, the relationship between taste and improved food quality perception has been well established and is likely to also extend to the broader fruit segment (Stiletto and Trestini 2021). Other attributes, such as the influence of products’ texture on quality perceptions, have not been as well documented but have also been found to influence food product quality perceptions positively (Symmank 2019). As an example, Bakke and Vickers (2011) reported that the roughness of bread influences quality perceptions positively. Similar observations have been made by Alonso, Paquin, and Mangin (2002) for fruits.

#### H7a

A positive perception of extrinsic quality cues enhances the overall food quality perception.

#### H7b

A positive perception of intrinsic quality cues enhances the overall food quality perception.

#### H7c

A positive perception of credence quality attributes enhances the overall food quality perception.

#### H7d

A positive perception of experience quality attributes enhances the overall food quality perception.

## Data collection and analysis

As no common understanding of the different products that belong to the category of NHLFPs exists so far (Loyer 2016; Oude Groeniger et al. 2017), designing a questionnaire focusing on NHLFPs in general might result in response bias. To mitigate that risk this research focuses on one specific NHLFP, namely blueberries. Blueberries were chosen as the case under research for the following reasons: First, blueberries are most regularly cited as belonging to the superfood category across news media channels in high-income societies (Butterworth et al. 2020). Second, blueberries fit, from a scientific as well as from a consumer point of view, the health benefits that NHLFPs possess in the eyes of the public (Schweiger and Haas 2020, 31f., 114). Third, news media reports have linked the increasing consumption of blueberries to their popularity across social media platforms. The recent development of a ‘blueberry social media trend’ is just one example of blueberries’ growing popularity and resulting marketing potential on social media platforms (Gläßer 2021; Koch 2019). In addition to this, Germany was chosen as an appropriate country case under research as the consumption level of blueberries (460 g p.a. and household in the year 2021) as well as retailers advertisement expenditures are high and growing fast (Gläßer 2021; Koch 2019; Wiedenroth and Otter 2021a). Furthermore, due to its high-income levels, the consumption of superfoods as NHLFPs is more likely to take place in Germany and, in fact, has already been observed for blueberries (Wiedenroth and Otter 2021b).

After a pre-test with 114 German consumers in January 2020, the collection of primary survey data took place from May to June 2020 through the online panel provider ‘respondi.’ The survey links were accessible only once, and quota requirements for the minimum age of 18 years were applied for ethical reasons. Furthermore, age groups and gender were applied in line with the German population characteristics. For quality reasons, participants had to consume fresh fruits at least once per week. The final data set contains 697 participants as 72 complete questionnaires were excluded from the overall participant list for quality reasons. The questionnaire first provided an information text for the participants and employed the necessary quota requirements. This was followed by queries about participants’ environmental consciousness (derived in part from do Paço et al. (2013) and Roberts (1996)) and their consumption motives for and involvement with fruits. Subsequently, consumers’ perception of different blueberry product dimensions and their familiarity with blueberries were elicited. Only then was consumers’ health awareness (derived from Gould (1990)) and social media affinity (derived from Rapp et al. (2013)) examined to prevent consumers’ bias. The questionnaire concluded with questions on the respondents’ socio-demographic characteristics using nominal scales while all the other sections employed ratio scales (5-point Likert scales ranging from ‘fully agree’ (+ 2) to ‘fully disagree’ (− 2)). A detailed presentation of the constructs and developed items is provided in Table 1. The extracted data set was analyzed using the partial least square structural equation modeling (PLS-SEM) method with the SmartPLS 3.3.2 software, while the descriptive statistics were analyzed through Windows IBM SPSS Statistics 26. PLS-SEM is well suited to investigate complex causal relationships as well as explorative research designs, as are present here (Hair et al. 2019). In particular, when testing a novel framework with complex relationships between many different constructs and indicators, Hair et al. (2019) emphasize the application of PLS-SEM analysis.

**Table 1:** Reliability of the research model Source: Authors’ own calculation

Indicator	Statement	AV^1^	SD^2^	CL^3^	CR^4^	AVE^5^
Construct ‘Social media involvement’					0.923	0.669
InvolvSM_1	I use social media to follow sales and promotions	− 1.18	1.135	0.742		
InvolvSM_2	I frequently use social media to inform myself about events that have taken place	− 0.79	1.224	0.854		
InvolvSM_3	People use social media to reach me	0.09	1.433	0.719		
InvolvSM_4	I often use social media to inform myself about upcoming events	− 0.5	1.352	0.855		
InvolvSM_5	I use social media to improve my relationship with different brands	− 0.76	1.287	0.855		
InvolvSM_6	Social media helps me a lot with improving my knowledge about companies that interest me	− 0.75	1.256	0.869		
Construct ‘Fruit-related desire to self-represent’					0.856	0.599
Motiv_2	I eat fresh fruits when I’m with friends	− 0.34	1.098	0.722		
Motiv_4	Often I eat fresh fruit directly before or after doing sports	− 0.39	1.254	0.732		
Motiv_5	Sometimes I consume fresh fruit to show my friends how health consciously I live	− 1.34	0.982	0.840		
Motiv_6	Sometimes I consume fresh fruit to show my family how health consciously I live	− 1.2	1.067	0.796		
Construct ‘Environmentalism’					0.904	0.611
Environmnt_1	I avoid buying products that have excessive packaging	0.91	0.996	0.730		
Environmnt_2	When there is a choice, I choose the product that causes the least pollution	0.84	1.003	0.767		
Environmnt_3	I make every effort to buy paper products made from recycled paper	0.58	1.049	0.814		
Environmnt_4	I use environmentally friendly soaps and detergents	0.28	1.15	0.800		
Environmnt_5	I remind members of my family and friends regularly not to buy some products that are harmful to the environment	− 0.31	1.223	0.798		
Environmnt_6	I try to buy products that can be recycled	0.73	0.963	0.779		
Construct ‘Health awareness’					0.921	0.625
Health_1	I reflect about my health a lot	0.53	1.062	0.797		
Health_3	I’m very self-conscious about my health	0.9	0.878	0.738		
Health_4	I’m constantly examining my health	0.38	1.082	0.768		
Health_5	I’m alert to changes in my health	0.56	0.951	0.691		
Health_7	I’m aware of the state of my health as I go through the day	0.34	1.042	0.852		
Health_8	I notice how I feel physically as I go through the day	0.57	1.033	0.836		
Health_9	I’m very involved with my health	0.05	1.139	0.840		
Construct ‘Age’			1.000		1.000	1.000
Age	In which year were you born (recoded into number of years)	49.69	16.97			
Construct ‘Income’					1.000	1.000
Income	Middle-income group (household income between 2500 to 2999 euros per month after taxes)	0.099	0.299	1.000		
Construct ‘Lean media’					0.771	0.633
TM_I	How often do you read daily and weekly newspapers?	1.4	0.49	0.911		
TM_II	How often do you read advertising brochures?	1.31	0.465	0.660		
Construct ‘Rich media’					0.767	0.530
SocialMedia_I	How often do you visit relations networks on the Internet (e.g., Facebook, LinkedIn, Xing)?	1.43	0.496	0.762		
SocialMedia_II	How often do you visit platforms for sharing pictures (e.g., Instagram, Flickr, Picasa)?	1.67	0.471	0.829		
SocialMedia_III	How often do you visit bookmarking websites (e.g., Pinterest, Digg, Reddit)?	1.82	0.383	0.566		
Construct ‘Search quality’					0.804	0.672
Observation_1	I touch the fresh fruit and examine it before I purchase it	0.45	1.04	0.847		
Observation_3	I smell the fresh fruit product before I purchase it	− 0.61	1.17	0.792		
Construct ‘Experience quality’					0.813	0.685
Experience_1	I’m very familiar with the different features of blueberries	0.12	1.072	0.812		
Experience_3	Often I use fresh blueberries for cooking or baking	− 0.76	1.127	0.843		
Construct ‘Blueberry credence quality cues’					0.827	0.615
Extrinsic2_3	Few pesticides are used for growing blueberries	− 0.09	0.789	0.741		
Extrinsic1_7	Compared with other fresh fruits, blueberries are often locally grown	− 0.26	0.97	0.777		
Extrinsic1_8	The production of blueberries is particularly environmentally friendly	− 0.07	0.749	0.831		
Construct ‘Blueberry experience quality’					0.843	0.642
Intrinsic5_4	Fresh blueberries taste sweet to light sourish	1.24	0.721	0.753		
Intrinsic5_7	Fresh blueberries feel firm upon touching them	1.07	0.805	0.852		
Intrinsic5_8	Fresh blueberries are characterized by a firm pulp	0.96	0.84	0.796		
Construct ‘Blueberry extrinsic quality cue’					0.826	0.614
Extrinsic1_9	Blueberries have well-known quality seals	− 0.54	0.943	0.818		
Extrinsic1_5	Producers of blueberries are well known	− 0.33	0.945	0.781		
Extrinsic2_5	On blueberry packaging, helpful product information can be found	0.05	0.867	0.750		
Construct ‘Blueberry intrinsic quality cue’					0.786	0.624
Intrinsic5_5	Fresh blueberries smell sweet	0.49	0.86	0.771		
Intrinsic5_6	Larger blueberries are more convenient than smaller ones	− 0.25	1.02	0.808		
Construct ‘Quality expectation’					0.822	0.699
WahQua2_1	Blueberries are of higher quality than other fresh fruits	− 0.19	0.905	0.827		
WahQua2_2	Blueberries that can be purchased in Germany are of high quality	0.63	0.772	0.844		

^1^ AV average value; ^2^ SC standard deviation; ^3^ CL construct loadings; ^4^ CR construct reliability; ^5^ AVE average variance extracted

The following constructs were queried on a 5-point Likert scale (from + 2 = fully agree to -2 = fully disagree): ‘Social media involvement,’ ‘Fruit-related desire to self-represent,’ ‘Environmentalism,’ ‘Health awareness,’ ‘Lean media,’ ‘Rich media,’ ‘Search quality,’ ‘Experience quality attributes,’ ‘Credence quality cues,’ ‘Extrinsic quality cue,’ ‘Intrinsic quality cue,’ and ‘Quality expectation.’

## Results

### Descriptive statistics

Overall, a data set containing 697 complete questionnaires with an average participant age of 49 years was compiled (see Table 2). This data set is representative of the German population for gender groups as it contains 51.2% females and 48.8% males.^4^ Furthermore, representativeness was achieved for almost all age groups as well as federal state residency. Only people living in North Rhine-Westphalia and Baden-Wuertemberg (17.5% and 9.5% compared with 21.6% and 13.3% in Germany) are underrepresented and people of the city states of Berlin and Hamburg (6.2% and 3.4% compared with 4.4% and 2.2% in Germany) are slightly overrepresented. Different categories of household income are not entirely representative of the German population as lower income groups are slightly overrepresented and higher income groups are slightly underrepresented in this data set. In addition, the share of participants who received a lower education, 18.2%, is underrepresented.^5^ The remaining categories, such as participants’ health awareness (19.2% of people smoke and 57.2% exercise regularly), their fruit consumption (64.1% of respondents consume fruits at least once per week), and the average time of 191 min that participants spend on the Internet each day can be considered representative of the population in Germany.

**Table 2:** Descriptive socio-demographic representation of the data sample. *Source* Authors’ calculation

	Sample^1^	German population^2^
*Gender [%]*		
	Female: 51.2	Female: 50.7
	Male: 48.8	Male: 49.3
*Age Ø [%]*		
18–24 years	9.2	7.58
25–34 years	15.8	12.81
35–44 years	15.6	12.36
45–59 years	22.1	22.68
60 years and older	37.3	28.76
*Federal state [%]*^*7*^		
Schleswig–Holstein	2.7	3.5
Hamburg	3.4	2.2
Bremen	0.9	0.8
Lower-Saxony	10.0	9.6
Mecklenburg-Vorpommern	1.9	1.9
Berlin	6.2	4.4
Brandenburg	3.0	3.0
Saxony-Anhalt	1.7	2.7
Saxony	6.0	4.9
Thuringia	2.6	2.6
Bavaria	16.6	15.8
Baden-Wuertemberg	9.5	13.3
North Rhine-Westphalia	17.5	21.6
Hessen	10.5	7.5
Rhineland-Palatinate	6.0	4.9
Saarland	1.4	1.2
*Income household after taxes [%]*		
	Under €900: 18.5	Less than €900: 7.9
	€900 to 1499: 19.5	€901–1500: 16.5
	€1500 to 1999: 17.9	€1501–2000: 14.9
	€2000 to 2499: 16.9	€2001–2600: 15.7
	€2500 to 2999: 9.9	€2600–3200: 11.6
	€3000 to 3499: 7.6	€3200 and more: 33.4
	€3500 and more: 9.6	
*Education*		
Lower	18.2	35.0
Middle	36.3	31.1
Higher	45.5	33.88
*Health [%]*		
Smoking cigarettes	19.2	17,53
Sport activity	57.2	56.9^5^
*Fruit consumption [%]*		
Daily	34.6	20.02
Multiple times per week	11.0	33.19
Once per week	18.5	7.57
Repeatedly within one month (but not every week)	14.1	7.19
Approximately once a month	12.8	0.73
Less than once a moth	1.4	1.33
Never	7.6	0.26
*Internet affinity*^*4*^		
Ø minutes online per day	191,28	196
Ø minutes on social media per day	41.5	79

^1^Data sample (*N* = 697)

^2^Values based on BMWi (2018); Frees and Koch (2019); Grobecker et al. (2018); Rabe (2021); Statistisches Bundesamt (2019, 2020, 2021); Techniker Krankenkasse (2019); WHO (2015)

^3^Lower education: no school leaving certificate/lower secondary school/primary school; middle education: secondary school, polytechnic school, master school; higher education: grammar school, university (the highest achieved, level of education had to be indicated)

^4^Corrected for unrealistic outliers

^5^Includes at least once per week

^6^Includes consumption of up to once per week

### PLS analysis: quality parameters of the measurement model

**Fig. 3:**
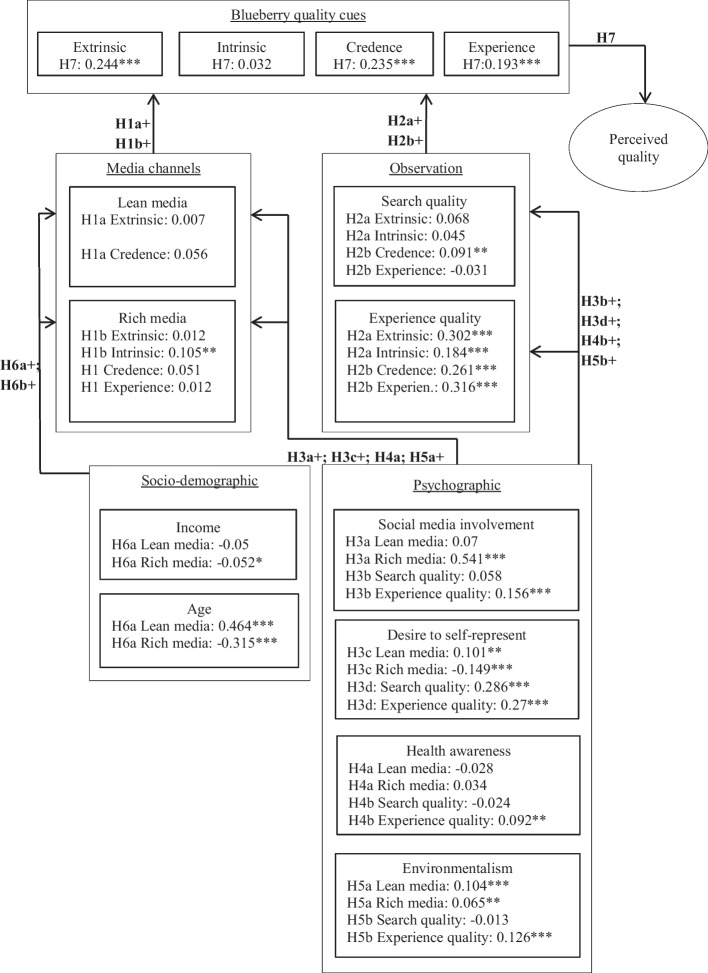
Determinants of NHLFP quality perception. *Source*: Authors’ own graphic adapted from Luning, Marcelis, and Jongen (2002) and Otter, Prechtel, and Theuvsen (2018). The numbers in parentheses represent path coefficients; level of significance: * = *p* ≤ 0.1, ** = *p* ≤ 0.05, *** = *p* ≤ 0.01

The results presented in Fig. 3 were tested for factor loadings, and almost all the items equaled or exceeded the threshold of ≥ 0.6. Only item three (‘How often do you visit bookmarking websites?’), belonging to the ‘rich media’ construct, showed a factor loading of 0.566. Internal consistency was confirmed by applying the composite reliability test (0.95 ≥ CR ≥ 0.7) (Fornell and Larcker 1981). To evaluate the convergent validity of the model, we analyzed the average variance extracted (AVE), and all the constructs met the desired threshold of AVE ≥ 0.5. In addition, all the items were placed below the preferred variance inflation factor (VIF ≤ 5) value. Next, the discriminant validity was evaluated by applying the heterotrait–monotrait ratio (HTMT). All the construct relations yielded a value below the threshold of HTMT ≤ 0.85 except the difference between ‘rich media’ and ‘social media involvement’ (HTMT of 0.856). This was to be expected but will be discussed further in Sect. 6. A detailed presentation of the latent variables and their respective correlations as well as discriminant validity is offered in Tables 1 and 3.

**Table 3:** Latent variables and respective correlations and discriminant validity. Source: Authors’ own calculation

	Age	Fruit-related desire to self-represent	Experience qualities	Blueberry credence qualities	Blueberry experience qualities	Blueberry extrinsic qualities	Blueberry intrinsic qualities	Health awareness	Income	Involvement SM	Lean media	Rich media	Perceived quality	Search qualities
Fruit-related desire to self-represent	− 0.298	0.774												
Environmentalism	0.033	0.326	0.782											
Experience qualities	− 0.082	0.417	0.271	0.828										
Blueberry credence qualities	− 0.114	0.376	0.249	0.289	0.784									
Blueberry experience qualities	0.095	0.003	0.098	0.312	0.135	0.802								
Blueberry extrinsic qualities	− 0.079	0.435	0.258	0.324	0.547	0.089	0.783							
Blueberry intrinsic qualities	− 0.147	0.304	0.075	0.212	0.315	0.261	0.305	0.79						
Health awareness	− 0.073	0.326	0.299	0.264	0.23	0.078	0.257	0.16	0.791					
Income	0.031	0.044	0.033	0.066	0.031	0.029	0.026	0.002	0.044	1				
Involvement SM	− 0.431	0.49	0.191	0.34	0.299	0.063	0.323	0.204	0.302	0.057	0.818			
Lean media	0.408	0.019	0.156	0.096	0.013	0.079	0.075	− 0.045	0.02	− 0.025	− 0.072	0.796		
Rich media	− 0.506	0.24	0.118	0.18	0.113	0.063	0.069	0.146	0.189	− 0.034	0.624	− 0.171	0.728	
Perceived quality	− 0.069	0.279	0.172	0.309	0.404	0.254	0.399	0.23	0.221	0.079	0.283	0.029	0.154	0.836
Search qualities	− 0.265	0.301	0.083	0.202	0.152	0.035	0.125	0.1	0.082	− 0.015	0.188	− 0.113	0.176	0.049

### PLS analysis: hypothesis testing

The raised hypotheses (see Fig. 2 and the *conceptual framework*) were analyzed regarding their *R*^2^ values and the presence of a significant relationship between different constructs. The recommendations for the necessary sample size with respect to the model design were followed, and the calculations applied the bootstrapping method with 5000 subsamples.

The derived adjusted *R*^2^ values yielded results of 0.203 for ‘lean media,’ 0.477 for ‘rich media,’ 0.224 for ‘experience qualities,’ and 0.094 for ‘search qualities.’ Blueberry characteristics showed adjusted *R*^2^ values of 0.111 for ‘extrinsic,’ 0.059 for ‘intrinsic,’ 0.095 for ‘credence,’ and 0.098 for ‘experience’ quality cues and attributes. Last, ‘perceived quality’ exhibited a value of 0.248. The presented results show that most *R*^2^ values, depending on the number of independent variables, lie below the threshold value of *R*^2^ ≥ 0.25, which Hair et al. (2019) interpreted as weak. Only the constructs ‘perceived quality,’ ‘lean media,’ and ‘rich media’ can be interpreted as moderate (Hair et al. 2019) (see Tables 1 and 3).

Starting with consumers’ psychographic characteristics, the model results show that consumers’ ‘social media involvement’ significantly influences their utilization of ‘rich media’ channels (*β* = 0.541***; H3a) as well as their blueberry-related ‘experience quality’ (*β* = 0.156***; H3b), while ‘lean media’ (*β* = 0.07) and ‘search quality’ (*β* = 0.058) are non-significantly influenced. Consumers’ desire to self-represent is shown to affect their ‘lean media’ utilization positively (*β* = 0.101**; H3c). Likewise, a positive effect on ‘search quality’ (*β* = 0.286***; H3d) as well as ‘experience quality’ (*β* = 0.27***; H3d) can be observed, while the opposite holds true for the usage of ‘rich media’ (*β* =  − 0.149***; H3c) channels. Similarly, heterogeneous effects are apparent for levels of consumers’ ‘health awareness,’ which impose a significant positive influence on consumers’ ‘experience quality’ (*β* = 0.092***; H4b) while revealing no significant impact on consumers’ ‘lean media’ (*β* =  − 0.028; H4a) and ‘rich media’ (*β* = 0.034; H4a) channel usage and ‘search qualities’ (*β* =  − 0.024; H4b). Conversely, ‘environmentalism’ affects ‘lean media’ (*β* = 0.104***; H5a), ‘rich media’ (*β* = 0.065**; H5a), and ‘experience quality’ (*β* = 0.126***; H5b) significantly positively. Consumers’ socio-demographics, show that ‘age’ has a positive impact on ‘lean media’ (*β* = 0.464***; H6a) while imposing a negative impact on ‘rich media’ (*β* =  − 0.315***; H6a). Likewise, consumers’ income influences their ‘rich media’ (*β* =  − 0.052*; H6a) utilization negatively.

Analyzing the different media channels, we observe that ‘lean media’ channels do not impose a significant influence on blueberry quality perception while ‘rich media’ channels only influence ‘intrinsic quality cues’ (*β* = 0.105**; H1b) significantly. Concerning consumers’ observational qualities, ‘search qualities’ influence the ‘credence quality cues’ (*β* = 0.091**; H2b) of blueberries significantly. In addition, ‘experience qualities’ with blueberries positively influence ‘extrinsic quality cues’ (*β* = 0.302***; H2a), ‘intrinsic quality cues’ (*β* = 0.184***; H2a), ‘credence quality cues’ (*β* = 0.261***; H2b), and ‘experience quality cues’ (*β* = 0.316***; H2b). In turn, ‘blueberry quality cues’ influence the ‘perceived quality’ of blueberries positively and most significantly, with ‘extrinsic quality cues’ constituting the largest significant effect (*β* = 0.244***; H7) followed by ‘credence quality cues’ (*β* = 0.235***; H7) and ‘experience quality cues’ (*β* = 0.193***; H7).

## Discussion

This research set out to investigate the effect that social media exerts on consumers’ quality perception of NHLFPs. Based on these findings, it was of further interest to discuss how these effects could accelerate short food supply chain formation in high-income countries like Germany. To address this topic, the food quality guidance model of Steenkamp (1989) and van Trijp and Steenkamp (2005) was modified, extended, and applied using PLS analysis, which was based on a data set of 697 respondents from Germany.

The original food quality guidance model was amplified in three ways: 1. it was extended with elements of media richness theory (Holt et al. 2015; Ledford 2012); 2. consumers’ product observation was subdivided into search and experience qualities following Nelson (1970); and 3. psychosocial consumer characteristics were integrated. Overall, the original part of the food quality guidance model within our overall conceptual framework has satisfactory explanatory power. Following Hair et al. (2019), the *R*^2^ value of the dependent variable, ‘perceived quality,’ shows a moderate influence, while the extrinsic, intrinsic, credence, and experience quality cues all impose a large and significant influence on perceived quality.

1. The integration of media richness theory is appropriate as we observe, just like Dunlop, Freeman, and Jones (2016) as well as Lipowski and Bondos (2018), and others, that rich and lean media sources impose different influences on the perception of different food quality attributes. We find ‘rich media’ to have a high and significant influence on intrinsic product perception while observing no influence from rich or lean media on any other product dimensions. This positive relationship is likely to result from the composition of the ‘rich media’ construct. Associated media platforms, like Instagram (SocialMedia II) and Pinterest (SocialMedia III), are designed primarily for sharing pictures and can be expected to have emphasized observable intrinsic product attributes, such as food color, most strongly, a circumstance that has been observed previously in gray literature sources (Green 2018; Southey 2019). Surprisingly, lean media sources, like advertisement brochures (TM_II, see Table 1), impose no significant influence on extrinsic product quality cues, such as the product price. Consequently, in the case of blueberries, lean media channels are less important for highlighting extrinsic product attributes as related findings (Lipowski and Bondos 2018; Smith et al. 2019) caused us to hypothesize in the conceptualization phase of this research. This is likely to be due to a dissonance between lean media sources, which in our case involve media types such as brochures (see Table 1) that often advertise reduced product prices, and the luxury dimensions of NHLFPs, for which consumers are less sensitive to price promotion marketing strategies.
2. Subdividing ‘product observation’ into the two subcategories search and experience qualities showed that search qualities impose much less influence on the quality perception of blueberries than experience qualities. Therefore, subdividing this latent variable in line with Nelsonʼs (1970) propositions helps to explain consumers’ quality perception process better and thus, allows for an appropriate modification of the food quality guidance model of Steenkamp (1989) and van Trijp and Steenkamp (2005). Search qualities influence the perception of credence attributes significantly and positively (see Fig. 3). Companies often aim to increase the observability of credence attributes prior to the purchasing decision through food labeling, as is the case, for example, for the country of origin (Otter, Prechtel, and Theuvsen 2018). Seemingly, this also holds true in the case of blueberries.
  Moreover, as blueberries are part of the NHLFP category, we know that corresponding consumer segments value luxury-related credence attributes highly (Wiedenroth and Otter 2021b). Following this, one can observe that the items that constitute the ‘credence quality cues’ construct in this research, namely the use of pesticides (Extrinsic2_3), locally grown (Extrinsic1_7), and environmental friendliness (Extrinsic1_8), were perceived by participants in other research projects as luxury food product dimensions (Hartmann, Nitzko, and Spiller 2016, 2017). Thus, consumers’ search-related food quality perception process is likely to build not only on credence but also on luxury-related credence attributes. This needs to be kept in mind in the discussion on the marketing of luxury-related credence attributes, such as the design of eco-friendly product packaging, which is gaining momentum among the greater NHLFP product category of superfoods (Ketelsen et al. 2020), or the carbon footprint labeling of food products (Meyerding et al. 2019).If they are easily visible, these labels could not only serve to inform consumers but could also, in the case of NHLFPs, be utilized for social comparison actions as they enhance food quality perceptions.
3. Consumers’ quality perception is primarily led by experience as ‘experience quality’ outweighs the influence of search qualities in the extent and significance of the path coefficients. In turn, experience quality is closely linked to consumers’ psychographic characteristics, supporting their integration into and this extension of the food quality guidance model in the first place. Furthermore, one can observe that the different motivations for NHLFP consumption proposed by Wiedenroth and Otter (2021b), namely health awareness and environmentalism as well as consumers’ desire for social comparison activities, all significantly influence experience quality. The high influence of experience quality on different product quality dimensions and the high influence of consumers’ psychographic characteristics on experience quality lead to two insights. Firstly, consumers are experience seeking when building their quality perception of blueberries. Secondly, their experience level is strongly led by the three different motives of NHLFP luxury consumption that Wiedenroth and Otter (2021b) presumed. The importance of consumers’ product experience in determining their purchasing behavior has been observed before (Frez Muñoz, Steenbekkers, and Fogliano 2016; Migliore et al. 2015). In the case of superfoods, which include NHLFPs, it has been emphasized that marketing strategies should try to develop product ‘stories’ more strongly. For example, many superfoods are deeply embedded into the regional culture and history of their country of origin. Emphasizing these product dimensions provides consumers with higher experience levels and leads to greater willingness to purchase superfoods (Strecker 2021). This trend is likely to be even more distinct among blueberries and NHLFPs in general because consumers could utilize such marketing content for socially comparable activities, preferably across social media platforms. In the case of blueberries, environmentalism and health awareness drive experience levels and ultimately food product quality perceptions. Consequently, marketing strategies should create product ‘stories’ that highlight these product attributes.

NHLFP consumers’ desire for engaging marketing content added to their high inclination toward SMM and socially comparable actions (Wiedenroth and Otter 2021b) might open up new alternative marketing channels to farmers. Generally, digitalization enables farmers and consumers to communicate more directly (Shepherd et al. 2020), and social media platforms provide an engaging communication tool at relatively lower costs (Elghannam et al. 2018, 2020). Farmers could take advantage of these current technological developments while addressing the observed blueberry quality perception process. Consumers value the opportunity to purchase from farmers as they associate this with locally grown products and more environmentally friendly, thus sustainable production practices (Cicia et al. 2021). As displayed before (see Fig. 3), this reflects the important luxury-related consumption motives of blueberries.

Besides obtaining an additional price premium from direct farm marketing, farmers could enhance this marketing channel by building on the current digitalization trends and advertise their products via social media platforms. This would be in line with the observed quality perception process of blueberries as ‘social media involvement’ significantly influences ‘experience quality,’ which in turn is the main driver of ‘perceived quality’ (see Fig. 3). Therefore, engaging with the final consumer through SMM is likely to be particularly profitable for farmers in the case of blueberries. This benefit possibly extends to most of the greater NHLFP product category as NHLFP consumer segments have been found to be highly receptive to SMM in general (Wiedenroth and Otter 2021b). In Germany, the focus of the current research, some farmers have already started establishing their own home pages and social media channels for marketing food products (Stache 2021; Zeisset and Fabry 2018). In doing so, farmers bypass the marketing monopoly of food retailers and other middlemen, which earns them the described price premium of direct farm marketing and leads to the development of SFSCs (Elghannam et al. 2018, 2020; Carbone 2017).

However, unlike other food products, the successful marketing of NHLFPs, such as blueberries, must also be highly engaging. This type of marketing content is often provided by larger companies, such as food retailers (Samoggia, Bertazzoli, and Ruggeri 2019), as SMM marketing itself is relatively cheap but engaging marketing content is frequently more cost intensive. This could overburden individual farmers. A solution, which is generally well established in the farming sector, would be the formation of closer horizontal ties to collective action as a means of sharing marketing production costs. This tendency already exists in the blueberry production sector as producer and retailer associations share marketing expenses (Park 2020). For SMM, obstacles arise from the induced development of SFSCs. Knierim et al. (2018) found that bottlenecks in the acceptance of digital innovation among farmers result from a lack of fit to the individual farm characteristics and the benefits from adopting new technologies not being noticeable enough. Both obstacles are likely to apply to digital NHLFP marketing as well. A lack of fit is crucial as NHLFPs’ luxury dimensions need to be advertised in correspondence to farm characteristics. For example, organically producing farmers might want to highlight their low use of pesticides, which fits the high environmental awareness of NHLFP consumers, while non-organic producing farmers might instead want to advertise the environmental benefits of locally produced food. Therefore, producers are, from an NHLFP marketing point of view, rather heterogeneous in characteristics and sharing marketing content is unlikely to be able to provide this level of detail and differentiation.

The benefit of technological adoption is crucial as consumers who engage with blueberry online marketing content do not necessarily purchase products at exactly the farm that has invested in this marketing content. Even if NHLFP marketing content in SMM is engaging, the two bottlenecks might lead to producer organizations highlighting general health- and luxury-related product attributes, which fit most farm characteristics of their members. This might lack the specificity of different NHLFP luxury dimensions in tune with individual farm characteristics that consumers demand. Such disincentives to collaborate could be overcome by farmers’ organizations providing customized digital marketing services instead of developing one common SMM strategy. Offering farmers a range of different engaging marketing tools that they can choose from in line with their farm characteristics would address both bottlenecks of technology adoption described by Knierim et al. (2018). For example, such marketing content could consist of pre-made online templates or cooking videos. Recent start-ups that provide meat producers with marketing templates and develop apps that allow for the creation of highly individual marketing content (Hufelschulte 2021) could be a benchmark for farmers’ associations to provide NHLFP product marketing services.

## Conclusion

SMM is an innovative but already well-established form of product marketing. In the case of food products, consumers can learn about different products and compare their activities easily across social media platforms, for which reason SMM is regarded as a promising tool for marketing NHLFPs, which has yet to unfold its full potential (Wiedenroth and Otter 2021b). Therefore, this research investigated the effects of social media on consumers’ quality perception of NHLFPs and their potential for accelerating SFSC formation in high-income countries, such as Germany. Media richness theory was integrated into the food quality guidance model (Steenkamp 1989; van Trijp and Steenkamp 2005) to uncover the SSM potential for companies along NHLFP supply chains. The resulting conceptual framework was examined using a data sample of 697 German blueberry consumers by applying the PLS-SEM method.

The research findings support the integration of the media richness theory into the food quality guidance model. They highlight the importance of consumers’ psychosocial characteristics for NHLFP quality perception, in particular their social media involvement, health, and environmental awareness, as well as their desire for socially comparable activities. For SMM of NHLFPs to be utilized successfully by retailers, the marketing content needs to be designed in accordance with these drivers and focus on providing engaging advertisement elements, which provide consumers with novel product-related experiences. The same holds true for farmers who want to circumvent oligopolistic structures in the supplier–buyer relationships with large retailers and engage in NHLFP SMM themselves. Besides providing engaging marketing content, their SMM must be tailor-made in accordance with their individual farm characteristics, fitting the NHLFP sustainability demands of the consumers. SFSCs provide opportunities for farmers as NHLFP consumers show a tendency to prefer direct engagement with producers. Horizontal collaboration might help farmers to reduce the individual costs of implementing and maintaining SMM, albeit not in the traditional form of marketing cooperatives. To preserve a competitive advantage through the differentiation strategy in geographical niches, SMM needs to be well aligned with individual farm characteristics. Farmers’ associations and agricultural consultancies could function as service providers following the example of some recent start-ups that provide online marketing templates that can be customized by individual farmers and help in developing their SSM strategies and implementation. Policy makers are left with the task of assisting farmers in executing innovative business models. This means assisting farmers in accessing higher SMM skills, for example, through subsidized training units, and ensuring sufficient internet access, which remains a bottleneck in faster digitalization in rural areas of some high-income countries, such as Germany (Shepherd et al. 2020).

This research is subject to some limitations. First, the sample is not fully representative across all income groups and education levels in Germany. This might have affected the results as consumers with high-income or low education levels, both groups that are slightly underrepresented in this research, might differ in their food consumption behavior, especially when it comes to luxury food products. Furthermore, the data collection took place during a time of high social restrictions in Germany due to the COVID-19 pandemic and other research has shown that the consumption patterns during this time were different from those in previous years (Busch et al. 2021; Wiedenroth and Otter 2021a).

Future studies should continue this research. According to this study, SMM, in the case of NHFLPs, offers farmers the opportunity to circumvent the oligopolistic structures in many supplier–buyer relationships with retailers in food supply chains. In response, retailers can increasingly focus on establishing their own sustainability labels (Gabot.de 2021), which correspond to the luxury dimensions of NHLFPs, and offer individual farmers SMM possibilities through their official websites (FruchthandelMagazin 2021). Even if this might reduce individual farmers’ costs of establishing and maintaining SMM, much of the price premium stemming from SMM might also remain with the retailers. This could inhibit farmers in generating profits from product innovations and impede their access to SMM revenue streams, which the current digitalization trends have opened in the first place. Therefore, future research should investigate how farmers perceive SMM channels led by food retailers and whether food retailers are already participating in a new form of vertical backward integration, fueled by the creation of SMM dependencies on farmers. Further analyses might shed more light on how farmers’ collaboration can develop the type of modifiable and highly engaging SMM marketing content discussed in this research.

## Data Availability

The data sets used and/or analyzed during the current study are available from the corresponding author on reasonable request.

## Appendices

See Tables 1, 2, 3.

See Figs. 1, 2, 3.

## Abbreviations

NHLFP: New healthy luxury food products
SMM: Social media marketing
SFSC: Short food supply chains
PLS: Partial least square
AVE: Average variance extracted
VIF: Variance inflation factor
HTMT: Heterotrait–monotrait ratio

## Footnotes

1 ‘NHLFP are traditional food products beneficial to a person’s health that are utilized as luxury products due to their above average product price and coexistence of health-related search and experience attributes’ (Wiedenroth and Otter 2021b). Superfoods displaying this combination of attributes, which can be utilized as luxury dimensions by the consumer, belong to the superfood subcategory NHFLP.

2 In the remainder of this paper SFSC are regarded as part of alternative food networks.

3 Whenever rich media channels are mentioned in this research, we refer to social media platforms only.

4 No participants identified themselves as members of a non-binary gender group.

5 Lower education: no school leaving certificate/lower secondary school/primary school.
